# Data, instance sets, and instances generator for the Hop-Constrained Minimum Spanning Tree problem, the Delay-Constrained Minimum Spanning Tree problem, and their bi-objective variants

**DOI:** 10.1016/j.dib.2023.109553

**Published:** 2023-09-07

**Authors:** Iago A. Carvalho, Amadeu A. Coco

**Affiliations:** a Department of Computer Science, Universidade Federal de Alfenas, Alfenas, Brazil; b ORKAD, CRIStAL, CNRS, Centrale Lille, Université de Lille, Lille, UMR 9189, France

**Keywords:** Multi-objective optimization, Bi-objective optimization, Constrained minimum spanning tree problems, Graphs, Optimization

## Abstract

This article proposes a benchmark instance generator for the Hop-Constrained Minimum Spanning Tree problem, the Delay-Constrained Minimum Spanning Tree problem, and their bi-objective variants. The generator is developed in C++ and does not uses external libraries, being understandable, easy-to-read, and easy-to-use. Furthermore, the generator employs five parameters that makes possible to generate personalized benchmark instances for these problems. We also describe 640 benchmark instances that were previously used in computational experiments in the literature. Lastly, we include raw results obtained from computational experiments with the described benchmark instances. We hope that the data introduced in this article can foster the development and the evaluation of new algorithms for solving constrained minimum spanning tree problems.

Specifications Table

**Table:** 

Subject	Decision Sciences

Specific subject area	Multi-objective optimization problems in graphs, most specifically bi-objective constrained minimum spanning tree problems
Data format	Raw, Analysed
Type of data	C++ File, CSV Tables, Text Files
Data collection	The data has been generated in-silico in a laboratory environment. It is useful to evaluate both exact and heuristic multi-objective optimization algorithms. The dataset can be separated into three pieces. The first is the instance generator, which is a C++ algorithm, used to generate instances for the Hop-Constrained Minimum Spanning Tree problem [1] and the Delay-Constrained Minimum Spanning Tree problem [2]. This instance generator makes use of 5 parameters to generate different instances. The second piece is the instances generated using the instance generator, which was employed in the paper [3]. The last piece is the raw data used to generate parts of Tables 1 and 2 and Figs. 4 and 5 of the paper [3].
Data source location	All data was sintetically generated in the Computational Intelligence Laboratory of the Computer Science Department of the Universidade Federal de Alfenas (UNIFAL). The university is located in Av. Jovino Fernandes de Sales, 2600 - Santa Clara, Alfenas - MG, 37133-840, Brazil. The information contained in the dataset has no geographic reference.
Data accessibility	Repository name: Data for "On solving bi-objective constrained minimum spanning tree problems" Data identification number: 10.17632/zck24s5wdf.2 Direct URL to data: doi:10.17632/zck24s5wdf.2
Related research article	Carvalho, I. A., & Coco, A. A. (2023). On solving bi-objective constrained minimum spanning tree problems. Journal of Global Optimization, 1-23. https://doi.org/10.1007/s10898-023-01295-8

## 1 Value of the data

• The dataset contains 320 instances for the Hop-Constrained Minimum Spanning Tree problem and other 320 instances for the Delay-Constrained Minimum Spanning Tree problem. These instances can be used as benchmark for these problems or for their bi-objective variant and were used as benchmark instances in [3].
• The dataset also contains the algorithm employed to generate the benchmark instances. This algorithm was developed in C++ and contain 5 parameters that can be used to customize the generated instance.
• There exists 4 *.csv* files containing the raw experimental results of [3]. These *.csv* files were employed to generate Tables 1 and 2 and Figs. 4 and 5 of the paper [3].
• The instances can be used to evaluate algorithms for the Hop-Constrained Minimum Spanning Tree problem, the Delay-Constrained Minimum Spanning Tree problem, or their bi-objective variants.
• The instance generator is useful to construct other instances for these problems. The generator is able to build small-, medium-, and large-sized instances in two different classic topologies of the literature.
• The instance generator can be adapted to generate instances for other constrained minimum spanning tree problems, such as the Degree-Constrained Minimum Spanning Tree problem [4] or the Minimum-Cost Bounded-Error Minimum Spanning Tree problem [5].

## 2 Objective

This paper describes an instance generator for the Hop-Constrained Minimum Spanning Tree problem, the Delay-Constrained Minimum Spanning Tree problem, and their bi-objective variants. Along with the instance generator, it also presents 640 benchmark instances, being 320 instances for each problem, which were employed in the experiments of [3]. The objective is to provide an adequate set of benchmark instances to evaluate algorithms for solving these problems.

## 3 Data description

**Fig. 1:**
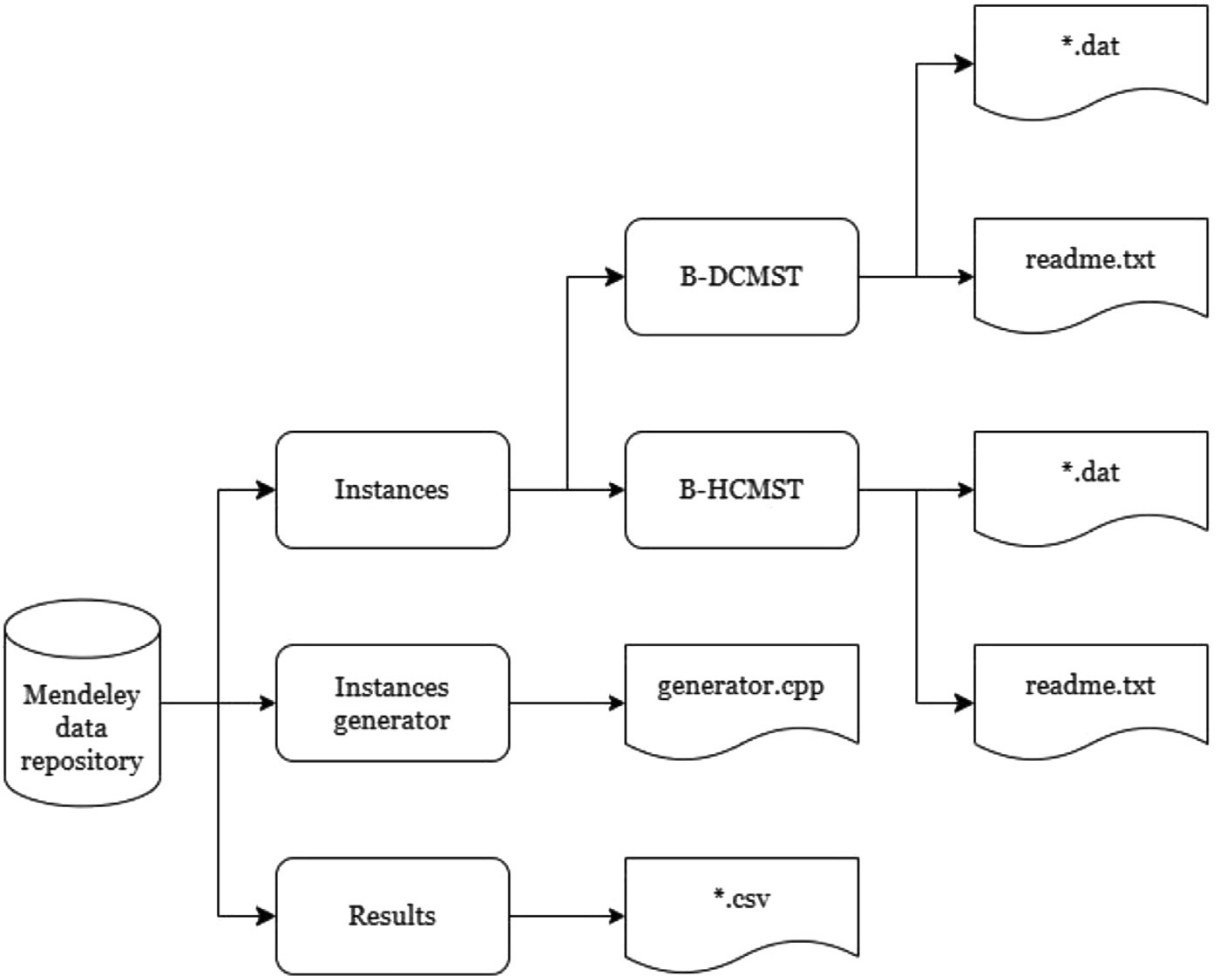
Organization of the Mendeley data repository.

The dataset is divided into three directories, as presented in Fig. 1. Directory “Instances generator” contains the instance generator algorithm developed in C++, directory “Instances” contains the benchmark instances generated by the above-mentioned generator algorithm and employed in [3], and directory “Results” contains the raw data used to construct Tables 1 and 2 and Figs. 4 and 5 of [3]. These directories and their files will be presented in details below.

### 3.1 Directory instances generator

This directory contains a single file denominated “generator.cpp”. This is a C++ algorithm that can be used to generate benchmark instances for the Delay-Constrained Minimum Spanning Tree problem (DCMST) and for the Hop-Constrained Minimum Spanning Tree problem (HCMST). These instances can also be used for their bi-objective variants, *i.e.*, the Bi-Objective Delay-Constrained Minimum Spanning Tree problem (B-DCMST) and the Bi-Objective Hop-Constrained Minimum Spanning Tree problem (B-DCMST).

The algorithm do not makes use of external libraries and can be easily compiled using any C++ compiler. For example, in Linux environments, one can compile the algorithm using the command *g++ generator.cpp -o generator*. Other compilers, such as *clang* or *Intel C++ compiler*, can also be used.

The compiled algorithm makes use of five parameters, as shown in Table 1. They need to be passed to the algorithm in the exact order they are displayed in Table 1. For example, in Linux environments, one could run the algorithm using the command *./generator 20 1 3 2 100*, whereas *20* represents the number of nodes in the instance, *1* denotes the instance's topology, 3 is the seed for the Mersenne Twister [6] pseudo-random number generator algorithm, *2* represents the chosen problem, and *100* is the maximum allowed delay for the DCMST instance (or B-DCMST instance). In the case that the parameters are not properly passed to the algorithm, a help message will be displayed for the user.

**Table 1:** Parameters employed by the benchmark instance's generator.

Parameter	Description	Possible values
size	Number of nodes of the benchmark instance	Natual numbers greater than 2
type	Instance topology	{1, 2}
seed	Seed for the pseudo-random number generator	Any integer number
problem	The problem for which the benchmark instance will be created	{1, 2}
delay	Maximum delay for the DCMST or B-DCMST	Natural numbers higher than 1

**Fig. 2:**
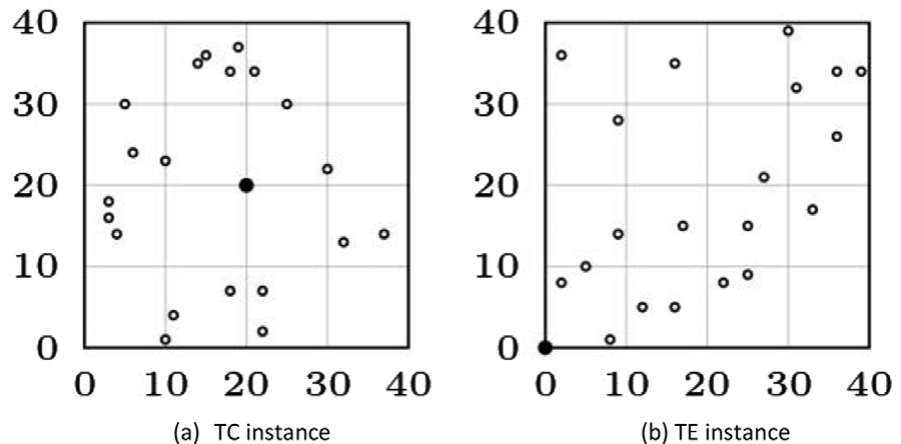
Illustrations of possible generated benchmark instances with 20 nodes. In these examples, the root node is drawn in black.
• **seed**: it should be an integer number.
• **problem**: two problems can be selected. The value 1 corresponds to a benchmark instance for the HCMST (or the B-HCMST), while the value 2 denotes a benchmark instance for the DCMST (or the B-DCMST).
• **delay**: it should be a natual number greater than 1. It will only be used in the cases that problem is set to 2. When problem is set to 1, this parameter will be discarded.

Every parameter has a predefined set of allowed values:

• **size**: it should be a natural number greater than 2.
• **type**: there exists two instance's topology, namely TC and TE. The value 1 denotes the TC instance, while the value 2 denotes the TE instance. Both instances topology are exemplified in Fig. 2. One may observe that the only difference between them is on the position of the root node. In TC instances, the root node is positioned in the center of the plane. On the other hand, in TE instances, the root node is positioned in the corner of the plane.

The generated instance will be a complete undirected graph. Every node (except the root) is randomly placed into a 40 × 40 plane using an uniform distribution. Furthermore, the edges’ weights are computed as the Euclidean distance between the nodes. For the DCMST (or B-DCMST) instances, the delay associated to every edge is computed randomly using an uniform distribution. The benchmark instance data is displayed for the user in the default output employed by the user.

The benchmark instance data is easy-to-read and to understand. In the first line, it displays, respectively, the number of nodes and the number of edges of the instance. Then, every remaining line denotes an edge. The first and second columns give the endpoints of the edge, while the third column shows the edge's weight. In the case of instances for the DCMST (or B-DCMST), a fourth column that denotes the delay of the edge also exists. One must observe that the root node is represented as the node zero.

#### 3.2 Directory instances

**Fig. 3:**
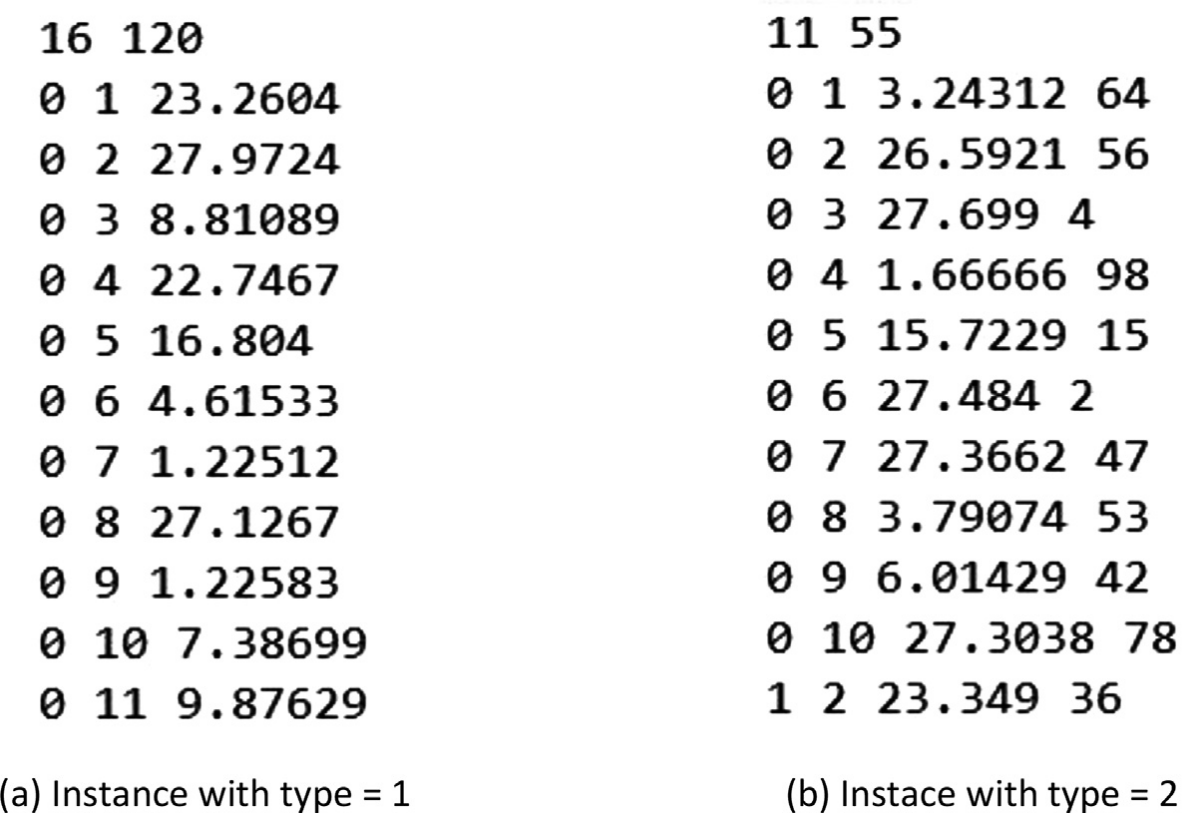
Excerpts from possible benchmark instances obtained from the generator algorithm.

This directory is divided into two sub-directories. The first, denominated as “B-DCMST”, contains the benchmark instances for the DCMST and for the B-DCMST along with a .txt file that describes the benchmark instance files. The second directory, denominated as “B-HCMST”, contains the benchmark instances for the HCMST and for the B-DCMST along with a .txt file that describes the benchmark instance files. All benchmark instances were employed in the computational experiments of [3]. Fig. 3(a) shows an excerpt from the instance *TC-16-7.dat* from the first sub-directory, while Fig. 3(b) shows an excerpt from the instance *TC-11-100-9.dat* from the second sub-directory.

For the “B-DCMST” directory, the instance name is given by a tuple *<a>-<b>-<c>.dat*. The value of *a* denotes the instance topology, *i.e*., TC or TE. Furthermore, the entry *b* gives the number of nodes in the benchmark instance. Besides that, the symbol *c* presents the seed passed to the pseudo-random number generator. This directory contains a total of 320 benchmark instances whose size varies from 10 to 25 nodes. It is possible to observe that exists 20 different instances of each size, which were created using distinct seeds for the pseudo-random number generator.

For the “B-HCMST” directory, the instance name is given by a tuple *<a>-<b>-<c>-<d>.dat*. The symbols *a, b*, and *c* denote the same parameters as described for the “B-DCMST” directory. In addition, the entry *d* gives the maximum delay of the benchmark instance. This directory also contains 320 benchmark instances whose size varies from 10 to 25 nodes. Similarly, there exists 20 different instances of each size that were created using different seed values for the pseudo-random number generator.

#### 3.3 Directory results

This directory contains 4 .csv files reporting the results obtained by the algorithms of [3] on the benchmark instances contained in the directory “Instances”. The files files “B-HCMST-cost.csv” and “B-HCMST-hop.csv” give the results of the computational experiments for the B-HCMST, while “B-DCMST-cost.csv” and “B-DCMST-delay.csv” report the computational results for the B-DCMST.

Every .csv file is composed of 5 columns. The first column denotes the benchmark instance topology, i.e., TC or TE. The second column gives the number of nodes in the instance, while the third column presents the seed used for the pseudo-random number generator. The fourth and fifth columns give the results of the algorithm. The fourth column reports the status of the algorithm, which can be “optimal” if an optimal solution was found, or “interrupted” if an optimal solution was not found in less than 10 hours. The fifth column report the time spent running the algorithm.

## 4 Experimental design, materials and methods

The benchmark instances contained in directory “Instances” were artificially generated using the C++ algorithm give in this dataset. The data of the .csv files of directory “Results” were obtained using two different implementations of the Augmented ε-constraints algorithm (AUG) [7]. Each .csv file give the result of a different implementation of AUG for a different problem. The raw data in files “B-HCMST-cost.csv” and “B-HCMST-hop.csv” were employed to generate Table 1 of [3], while the raw data in files “B-DCMST-cost.csv” and “B-DCMST-delay.csv” were used as source for computing Table 2 of [3]. The raw data of these files were also employed to plot Figs. 4 and 5 of [3].

## Limitations

The data reported in directory “Results” is not sufficient for reconstructing Tables 1 and 2 of [3] as the size of the Pareto-sets are not given in this repository.

## Ethics statement

The authors have read and follow the ethical requirements for publication in Data in Brief and confirm that the current work does not involve human subjects, animal experiments, or any data collected from social media platforms.

## CRediT authorship contribution statement

**Iago A. Carvalho:** Conceptualization, Data curation, Writing – original draft. **Amadeu A. Coco:** Writing – review & editing.

## Data Availability

Data for “On solving bi-objective constrained minimum spanning tree problems” (Original data) (Mendeley Data).
